# Association of Blood‐based DNA Methylation Markers with Cognition in Alzheimer’s Disease

**DOI:** 10.1002/alz.094863

**Published:** 2025-01-09

**Authors:** Kok Pin Ng, Ling Wang, Rajkumar Dorajoo, Marie Loh, Adeline Su Lyn Ng, Shahul Hameed, Simon Kang Seng Ting, Nagaendran Kandiah, Jianjun Liu

**Affiliations:** ^1^ Department of Neurology, National Neuroscience Institute, Singapore, Singapore, Singapore Singapore; ^2^ Duke‐NUS Medical School, Singapore Singapore; ^3^ Genome Institute of Singapore, Agency for Science, Technology and Research, Singapore, Singapore, Singapore Singapore; ^4^ Lee Kong Chian School of Medicine, Nanyang Technological University, Singapore City, Singapore, Singapore Singapore; ^5^ Genome Institute of Singapore (GIS), Agency for Science, Technology and Research (A*STAR), Singapore City, Singapore, Singapore Singapore; ^6^ Department of Epidemiology and Biostatistics, School of Public Health, Imperial College London, London, UK, London United Kingdom; ^7^ National Neuroscience Institute, Singapore Singapore; ^8^ National Neuroscience Institute, Singapore General Hospital, Singapore Singapore; ^9^ Lee Kong Chian School of Medicine, Nanyang Technological University, Singapore Singapore

## Abstract

**Background:**

DNA methylation is an epigenetic change characterized by the addition of methyl groups to DNA, typically in the cytosine‐ phosphate‐guanine (CpG) nucleotide base pairings. Given that DNA methylation alterations are shown to be associated with Alzheimer’s Disease (AD) pathology in autopsied brains, blood‐based DNA methylation changes are increasingly being studied as a potential peripheral biomarker for AD. However, the role of blood‐based DNA methylation changes as a marker of cognitive impairment in AD remains unclear. In this study, we aim to identify the blood‐based DNA methylation signatures that are associated with worse cognitive performance and brain atrophy in AD individuals.

**Methods:**

277 AD patients of Chinese ethnicity were recruited from a tertiary memory clinic (National Neuroscience Institute, Singapore). All participants underwent the Montreal Cognitive Assessment (MoCA) test and blood collection. CpG probe methylation was measured using the Illumina Infinium MethylationEPIC BeadChip. A subset of participants (n = 218, 78.7%) underwent MRI brain scan and medial temporal lobe atrophy (MTA) score was measured visually. Regression analysis evaluated the associations of each methylation marker with MoCA and MTA scores, corrected for age, gender and years of education.

**Results:**

We identified two methylation markers (cg11103255 and cg10845701) to be associated with MoCA scores. Hypermethymation at the cg11103255 site was associated with lower MoCA scores (p = 2.86e‐07) while hypomethylation at the cg10845701 site was associated with lower MoCA scores (p = 8.18e‐07). Furthermore, hypomethylation at the cg10845701 site was associated with higher MTA scores. cg11103255 is mapped to the Microtubule Crosslinking Factor 1 (MTCL1) gene, a protein coding gene that enables microtubule binding activity. cg10845701 is mapped to the Solute Carrier Family 44 Member 5 (SLC44A5) gene, a protein coding gene that enables transmembrane transporter activity. While the role of MTCL1 gene on AD is not known, SLC44A5 was previously reported to be associated with brain atrophy in an AD GWAS.

**Conclusion:**

Our findings demonstrate the potential role of blood‐based DNA methylation markers as measures of worse cognitive performance among AD. Further longitudinal studies in an independent cohort are needed to validate these findings.

